# Oxidative balance score and the potential for suffering rheumatoid arthritis: a cross-sectional study

**DOI:** 10.3389/fimmu.2024.1454594

**Published:** 2024-11-01

**Authors:** Yimin Zhang, Hao Yu, Jianfei Fu, Renjie Zhuo, Jin Xu, Liya Liu, Manyun Dai, Zhen Li

**Affiliations:** ^1^ School of Public Health, Health Science Center, Ningbo University, Ningbo, China; ^2^ Department of Urology, The Second Affiliated Hospital of Nanjing Medical University, Nanjing, China; ^3^ Department of Urology, The Second Clinical Medical College of Nanjing Medical University, Nanjing, China; ^4^ Department of Medical Records and Statistics, The First Affiliated Hospital of Ningbo University, Ningbo, China

**Keywords:** oxidative balance score, rheumatoid arthritis, NHANES, oxidative stress, nutritional status

## Abstract

**Objective:**

Our study was conducted to explore the link between oxidative balance score (OBS) and rheumatoid arthritis (RA).

**Methods:**

A total of 21,415 participants were included in our research from five cycles (2011–2012, 2013–2014, 2015–2016, 2017–2018, and 2017–2020) of the National Health and Nutrition Examination Survey (NHANES). Moreover, 20 elements related to diet as well as lifestyle were combined to calculate OBS. The relationship between OBS and RA was assessed by employing multivariable regression analysis, and further exploration was carried out through subgroup analysis, restricted cubic spline analysis, and sensitivity analysis. Multiple covariates were selected to adjust the model for more robust results.

**Results:**

In our cross-sectional study, a higher OBS has a protective effect on the development of RA (OR = 0.98, 95% CI: 0.97 to 0.99). In contrast to individuals aged ≥60, the result is more prominent in the population aged 20–60 (OR = 0.97, 95% CI: 0.96 to 0.98). Marital status appears to introduce interference in the relationship between OBS and RA, and unmarried individuals exhibited different outcomes (OR = 1.02, 95% CI: 0.99 to 1.04) compared to others. The positive influence of OBS was more evident in patients with chronic kidney disease and cardiovascular disease, while it was stronger in individuals without diabetes and liver disease.

**Conclusion:**

A higher OBS correlates with a reduced odd of RA. Further studies are needed to shoot more sights on improving dietary habits and lifestyles to gain proper OBS and explore whether OBS can be one of the measurements utilized to measure the risk of RA.

## 1 Introduction

Rheumatoid arthritis (RA) is a prevalent inflammatory disorder impacting the joints chiefly. As an autoimmune disease, it can also lead to damage of multiple organ systems outside the joints, including the cardiovascular system, liver, kidney, and so on (1, 2). Today, RA impacts around one in every 200 adults of the global population, with a greater occurrence rate among women. However, the precise cause of RA still remains uncertain; both genetic and environmental factors may play a role in the development of this disease (3). A previous study proclaimed that elevated levels of reactive oxygen species (ROS) is intimately associated with RA. A variety of oxidants and antioxidants in the human body are bound to play their roles in RA, which provide some ideas for people to prevent it and measure or improve the quality of life of RA patients (4).

Many studies have investigated how adopting healthy eating habits can act as an efficient approach to lower the likelihood of RA. Research findings suggested that the addition of antioxidants could potentially serve as a beneficial complementary approach in reducing oxidative stress in individuals with RA, and the use of zinc and selenium supplementation has been employed for many years in the prevention of RA remission (5). However, according to some research, it seemed that the intake of some antioxidants does not improve RA (6). Different scientists have different results on the effects of alcohol on RA (7, 8), but the effects of smoking, overweight, and unhealthy lifestyle on RA seemed to be certain according to the available studies (9).

The oxidative balance score (OBS) is developed to comprehensively evaluate the oxidative and antioxidant condition in the human body. It integrates multiple nutrient diets and various lifestyles, and generally an elevated OBS indicates a reduced pro-oxidant burden (10). Currently, an increasing number of epidemiological research are trying to find the correlation between OBS and certain prevalent illnesses. Some studies found that higher OBS scores are associated with a reduced prevalence of cancers (11). Similar negative relationships can also be found in OBS with diabetes (12) and depression (13), and it appears to be more pronounced in women. According to a study by Wang et al., OBS can also be employed to measure all-cause mortality and CVD death (14).

Each individual has his own dietary habit and lifestyle, which can be either beneficial or harmful factors for RA. By selecting the data from the National Health and Nutrition Examination Survey (NHANES), we utilize the OBS scoring system to comprehensively evaluate these factors. We aim to conduct the first systematic assessment of their influence on RA and the potential implications involved. We hope that this can provide insights for future prevention, diagnosis, and treatment of RA.

## 2 Materials and methods

### 2.1 Data sources and study population

**Figure 1:**
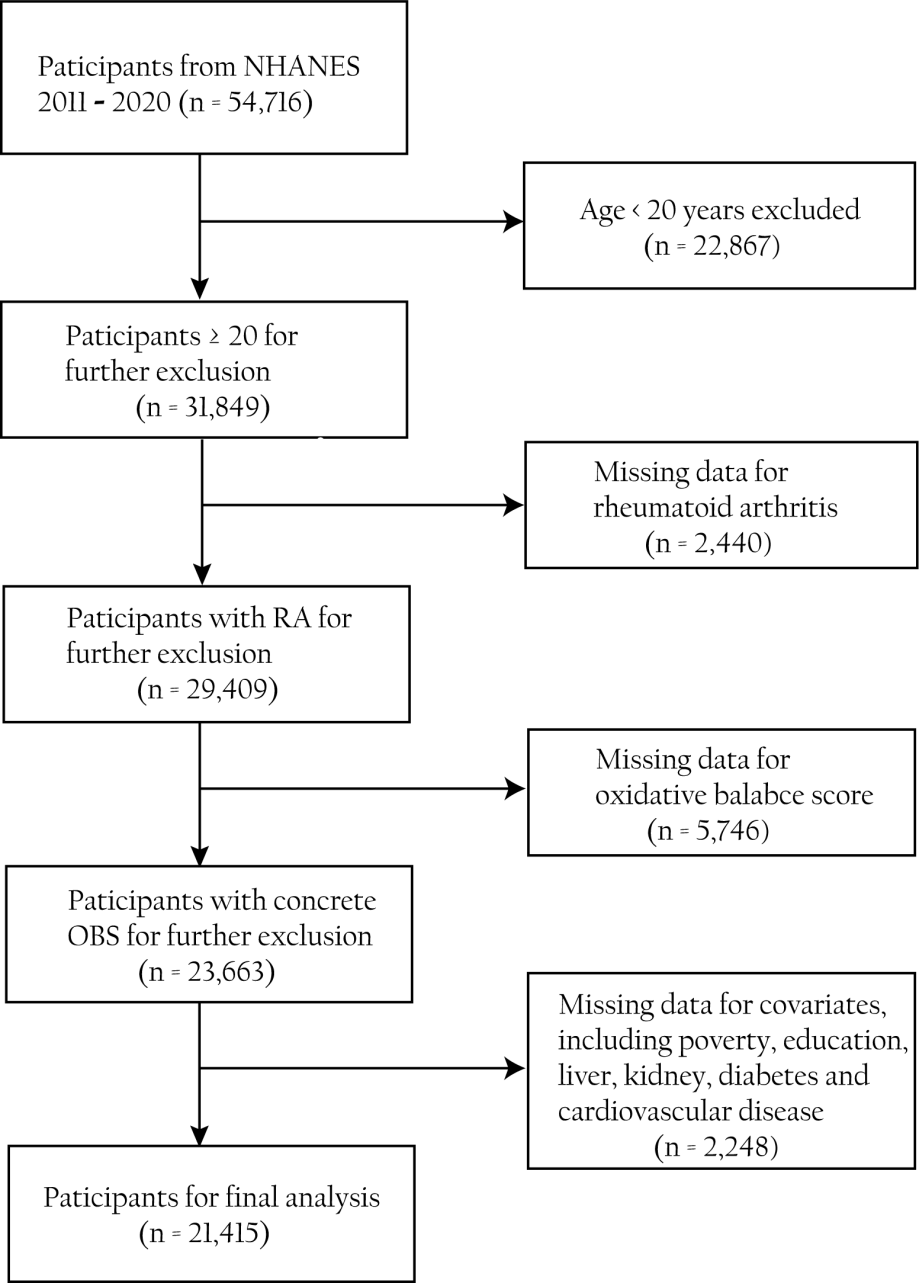
Flow diagram of the study selection process.

By collecting data on participants’ health, nutrition, lifestyle, and other aspects, the NHANES uses a stratified, multistage probability sampling way to evaluate the health and nutrition status of the American population (15). It is an important project that holds a significant value in understanding the health issues of the American population. From the study, 54,716 individuals were recruited from five cycles (2011–2012, 2013–2014, 2015–2016, 2017–2018, and 2017–2020) of the NHANES. We eliminated non-standard information involving (1) participants above 20 years old (*n* = 22,867), (2) participants who lack questionnaire information regarding RA (*n* = 2,440), (3) participants who lack dietary or laboratory information in the components of OBS (*n* = 5,746), and (4) incomplete covariates information on participants (*n* = 2,248). The elaborate flowchart outlining our work is displayed in **Figure 1**.

### 2.2 Diagnosis of RA

The evaluation of RA was established through a self-report questionnaire. The questionnaire included inquiries such as “Have you ever been diagnosed with arthritis by a doctor or other health professionals?” and “What specific type of arthritis did you have?” Respondents who answered “Yes” in the first question and identified their condition as “rheumatoid arthritis” in the second question were categorized into the RA group, while individuals who expressed a negative attitude toward the first question or choose other forms of arthritis were excluded from the non-RA group.

### 2.3 Computation of OBS

The computation of OBS for each individual was based on information from 16 dietary and four lifestyle elements, which can also be categorized as 15 antioxidants and five pro-oxidants (**Supplementary Table S1**). Specific calculation standards and methods were obtained from previous articles (16). It should be noted that during the calculation process, we need to first collect the relevant data from the physical activity questionnaire (PAQ). Then, the PAQ will be converted into metabolic equivalent (MET) based on the following formula: PA (MET-min/wk) = MET × weekly frequency × duration of each PA (17). Concrete data can all be obtained from NHANES. We use the serum cotinine concentration from laboratory data in NHANES as a substitute for smoking levels (16), and data on dietary fiber, carotene, riboflavin, niacin, calcium, magnesium, zinc, total folate, vitamins (B6, B12, C, and E), copper, selenium, total fat, iron, and alcohol are in the total nutrient intake interview. BMI can also be directly selected from examination data. There are gender differences in the scoring criteria for each indicator, and the scoring for intake of oxidants and antioxidants is also different.

### 2.4 Covariates data

In addition to common demographic covariates involving age, sex, race, educational, marital, and family poverty–income ratio (PIR), we also obtained information on kidney, diabetes, cardiovascular disease (CVD), and liver disease via standardized questionnaires. These possible variables may also cause potential interference to our result. Age was classified into two groups (20–60 and ≥60), and PIR was segmented into three groups (≤1.3, 1.3–3.5, and >3.5), which means low, medium, and high level, respectively (18). Chronic kidney disease was explained as urine albumin/creatinine ratio >30 mg/g or estimated eGFR ≤60 mL/min/1.73 m^2^ (19). The diagnostic criteria for diabetes included self-reported diagnosis by a doctor and ongoing oral treatment with diabetes medication. CVD was composed of five independent questions, and any positive answer from the participants was confirmed as CVD. Liver disease was based on the self-reported questionnaire from NHANES. More detailed covariate information can be viewed in **Supplementary Table S2**.

### 2.5 Statistical analysis

Every analysis was conducted using R (version 4.3.2), and statistical significance was defined as two-tailed *p <*0.05. Categorical or continuous variables were separately demonstrated by mean ± standard deviation (SD) and percentage (%), and *t*-test or chi-square test was utilized to analyze them as well. Based on the characteristics of the participants, OBS was categorized into quantiles: quartile 1 (Q1): [3, 12], quartile 2 (Q2): (12, 18], quartile 3 (Q3): (18, 24], and quartile 4 (Q4): (24,37], and quantile 1 was chosen as the reference. Four multivariate logistic regression models were developed to assess the association between OBS and the occurrence of RA. Model 1 was a basic model for no adjustments. Model 2 was adjusted for age and sex. Model 3 included adjustments for age, sex, race, education, marital status, and PIR. Model 4 further adjusted for age, sex, race, education, marital status, PIR, CKD, diabetes, CVD, and liver disease. Odds ratio (OR) and 95% confidence interval (95% CI) were used to present the results.

Subgroup analyses were performed with the presentation of forest plots, and interaction tests were chosen to examine the relevant statistical significance. Restricted cubic splines (RCS) analysis was employed to illustrate the nonlinear correlation between OBS and RA through the value of *p*-nonlinear. A sensitivity analysis was performed by successively omitting each of the 20 components of OBS to examine the robustness of our findings.

## 3 Results

### 3.1 Descriptive statistics

The prevalence of RA and the characteristics of the overall 21,415 participants in the 2011–2012, 2013–2014, 2015–2016, 2017–2018, and 2017–2020 cycles of the NHANES are exhibited below. A total of 1,259 individuals were diagnosed as RA (proportion, 5.9%), and 20,156 individuals were non-RA (proportion, 94.1%). The age group of 20–60 has the largest population (proportion, 68.7%), but in contrast to the non-RA group (proportion, 29.7%), RA people aged 60 and above have a higher proportion (proportion, 57.6%). In addition, the incidence rate of CKD, diabetes, CVD, and liver diseases in the RA group is obviously higher. Overall, a significant difference can be found in RA and non-RA groups of relevant characteristics mentioned in **Table 1**.

**Table 1:** Characteristics of participants aged 20–80 by RA in the US.

Characteristics	Overall (*n* = 21,415)	RA (*n* = 1,259)	Non-RA (*n* = 20,156)	*p*-value
Age, *n* (%)	48.7 ± 17.4	60.2 ± 13.1	48.0 ± 17.4	<0.001
20–60	14,705 (68.7)	534 (42.4)	14,171 (70.3)	
≥60	6,710 (31.3)	725 (57.6)	5,985 (29.7)	
Sex, *n* (%)				<0.001
Male	10,432 (48.7)	549 (43.5)	9,883 (49.0)	
Female	10,983 (51.3)	710 (56.5)	10,273 (51.0)	
Race, *n* (%)				<0.001
Mexican American	2,760 (12.9)	150 (11.9)	2,610 (12.9)	
Other Hispanic	2,125 (9.9)	131 (10.4)	1,994 (9.9)	
Non-Hispanic White	8,413 (39.3)	445 (35.3)	7,968 (39.5)	
Non-Hispanic Black	4,729 (22.1)	421 (33.4)	4,308 (21.4)	
Other race	3,388 (15.8)	112 (9.0)	3,276 (16.3)	
Education, *n* (%)				<0.001
Below high school	3,937 (18.4)	328 (26.1)	3,609 (17.9)	
High school	4,840 (22.6)	322 (25.6)	4,518 (22.4)	
Above high school	12,638 (59.0)	609 (48.3)	12,029 (59.7)	
Marital status, *n* (%)				<0.001
Married/cohabiting	12,833 (59.9)	691 (54.9)	12,142 (60.2)	
Widowed/divorced/separated	4,446 (20.8)	445 (35.3)	4,001 (19.9)	
Never married	4,136 (19.3)	123 (9.8)	4,013 (19.9)	
PIR, *n* (%)	2.0 ± 0.8	1.8 ± 0.8	2.0 ± 0.8	<0.001
≤1.3	6,531 (30.5)	523 (41.5)	6,008 (29.8)	
1.3–3.5	8,083 (37.7)	443 (35.2)	7,640 (37.9)	
>3.5	6,801 (31.8)	293 (23.3)	6,508 (32.3)	
CKD, *n* (%)				<0.001
Yes	5,775 (27.0)	519 (41.2)	5,256 (26.1)	
No	15,640 (73.0)	740 (58.8)	14,900 (73.9)	
Diabetes, *n* (%)				<0.001
Yes	2,147 (10.0)	234 (18.6)	1,913 (9.5)	
No	19,268 (90.0)	1,025 (81.4)	18,243 (90.5)	
CVD, *n* (%)				<0.001
Yes	2,207 (10.3)	316 (25.1)	1,891 (9.4)	
No	19,208 (89.7)	943 (74.9)	18,265 (90.6)	
Liver disease, *n* (%)				<0.001
Yes	928 (4.3)	88 (7.0)	840 (4.2)	
No	20,487 (95.7)	1,171 (93.0)	19,316 (95.8)	

Continuous variables were presented as mean ± standard deviation (SD). Categorical variables were presented as *n* (%).

RA, rheumatoid arthritis; PIR, family poverty–income ratio; CKD, chronic kidney disease; CVD, cardiovascular disease.

We also divided 21,415 participants into four groups according to OBS. The baseline traits of participants categorized by OBS quartiles are displayed in **Supplementary Table S3**. Younger individuals with higher levels of education and PIR were relatively with greater OBS. It was worth mentioning that the Q1 group exhibited the highest level of various diseases, while the Q4 group showed the lowest levels among the population.

### 3.2 Association of OBS with RA

After conducting a logistic regression analysis, the correlation between OBS and RA is exhibited in **Table 2**. A total of results consistently indicated a positive relationship between higher OBS scores and a reduction in RA risk. In the unadjusted model (model 1), with each OBS score increasing, a 4% lower odd of RA was displayed (OR = 0.96, 95% CI: 0.96 to 0.97). After adjusting all covariates, the association was slightly attenuated but still held positive statistical significance (OR = 0.98, 95% CI: 0.97 to 0.99). Comparing to Q3 and Q4 in model 1, we observed that the risk reduction was stronger in Q2, with a 5% lower odd of RA in each increment of OBS (OR = 0.95, 95% CI = 0.93 to 0.97). However, with the confounding factors adjusted gradually, the differences narrowed and displayed a 3% lower odd of RA in each increment of all quantiles in OBS.

**Table 2:** Relationship between OBS and RA in the US.

	Model 1		Model 2		Model 3		Model 4	
	OR (95% CI)	*p*-value	OR (95% CI)	*p*-value	OR (95% CI)	*p*-value	OR (95% CI)	*p*-value
Continuous	0.96 (0.96, 0.97)	<0.001	0.97 (0.96, 0.98)	<0.001	0.98 (0.97, 0.98)	<0.001	0.98 (0.97, 0.99)	<0.001
Q1	1.00 (ref)		1.00 (ref)		1.00 (ref)		1.00 (ref)	
Q2	0.95 (0.93, 0.97)	<0.001	0.95 (0.93, 0.97)	<0.001	0.96 (0.94, 0.98)	<0.001	0.97 (0.94, 0.99)	0.003
Q3	0.96 (0.95, 0.97)	<0.001	0.97 (0.95, 0.98)	<0.001	0.97 (0.96, 0.98)	<0.001	0.97 (0.96, 0.99)	<0.001
Q4	0.96 (0.95, 0.97)	<0.001	0.97 (0.96, 0.98)	<0.001	0.97 (0.96, 0.98)	<0.001	0.97 (0.97, 0.98)	<0.001

Model 1, no adjustment for covariates; model 2, adjustments for age and gender; model 3, adjustments for age, gender, race, education, marital, and PIR; model 4, adjustments for age, gender, race, education, marital, PIR, CKD, diabetes, CVD, and liver disease.

### 3.3 Subgroup analysis

**Figure 2:**
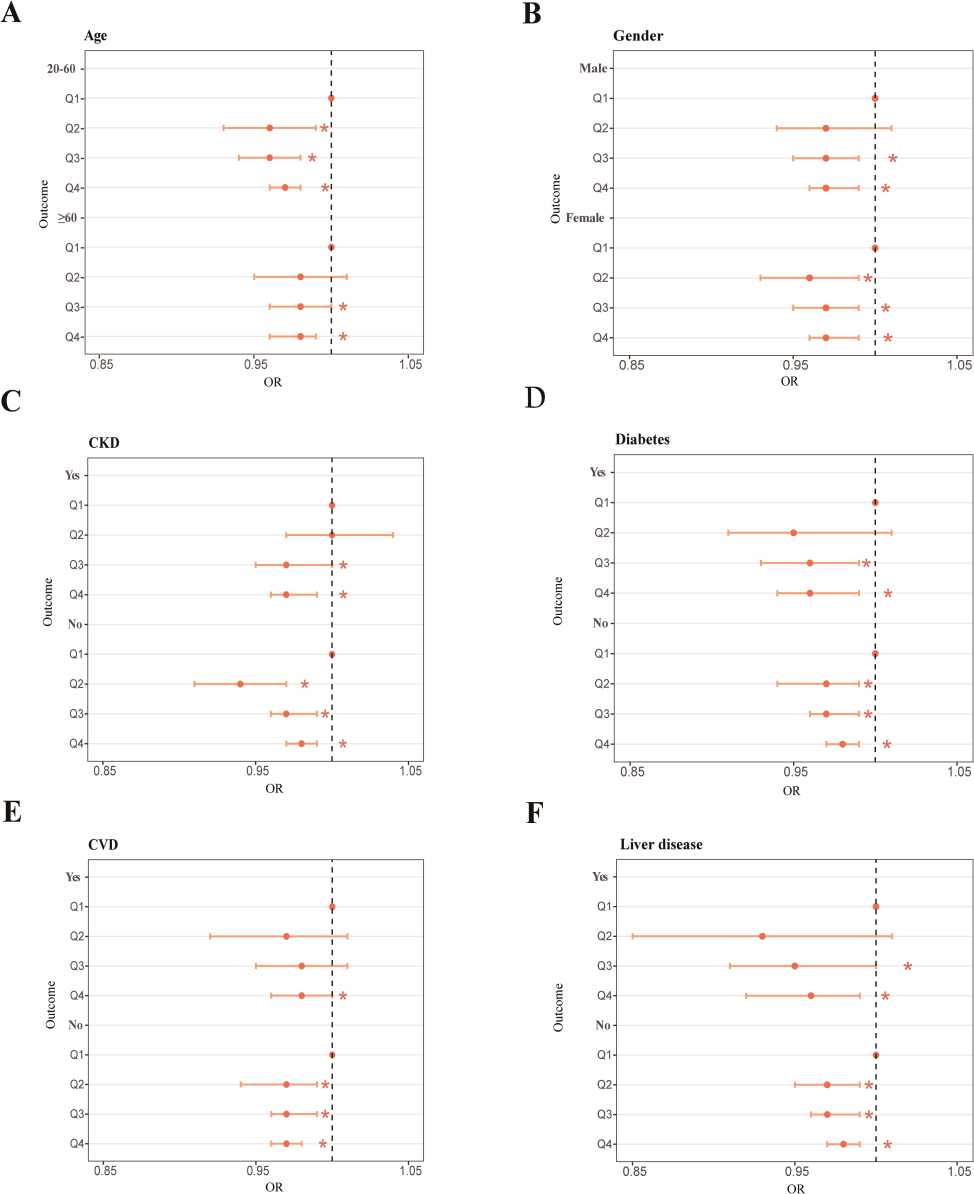
Forest plot of the age subgroup **(A)**, forest plot of the gender subgroup **(B)**, forest plot of the CKD subgroup **(C)**, forest plot of the diabetes subgroup **(D)**, forest plot of the CVD subgroup **(E)**, forest plot of the liver disease subgroup **(F)**. *p < 0.05.

We utilized the full-adjustment model (model 4) and a conducted subgroup analysis as well as interaction tests across 10 categories, examining different age ranges, gender, race, education, marital status, PIR, and four common types of diseases. Partial outcomes including age, gender, and four diseases are presented in **Figure 2**. The others are shown in **Supplementary Figure S1**. Contrasted with elderly individuals aged ≥60 (Q4: OR = 0.97, 95% CI: 0.96 to 0.98), it was evident that the impact of OBS on RA was more prominent among younger individuals aged 20–60 (Q4: OR = 0.98, 95% CI: 0.96 to 0.99) (**Figure 2A**). The effect of OBS on RA appeared to be less different between male and female individuals (**Figure 2B**). When examining diabetes (**Figure 2D**) and liver disease (**Figure 2F**), the negative correlation in individuals with each ailment exceeded that in the healthy group. However, the CKD (**Figure 2C**) and CVD (**Figure 2E**) subgroups displayed a stronger association between OBS and RA in healthy individuals. About race, the subgroup of non-Hispanic White and other race suggested a closer and negative link among all quantiles of OBS, while non-Hispanic Black exhibited a positive correlation in Q2 (OR = 1.04, 95% CI: 1.01 to 1.08) (**Supplementary Figure S1A**). Participants who completed education levels above high school (**Supplementary Figure S1B**), are married (**Supplementary Figure S1C**), and have PIR ranging from 1.3 to 3.5 (**Supplementary Figure S1D**) all exhibit a decrease in RA risk as OBS increases in all quantiles. However, we noticed that among unmarried individuals, the increase in OBS is associated with an increase in RA risk (**Supplementary Figure S1C**). Statistical significance was observed in the race (*p* for interaction = 0.034) and marital status (*p* for interaction = 0.032) subgroups of OBS.

### 3.4 Restricted cubic spline analysis

**Figure 3:**
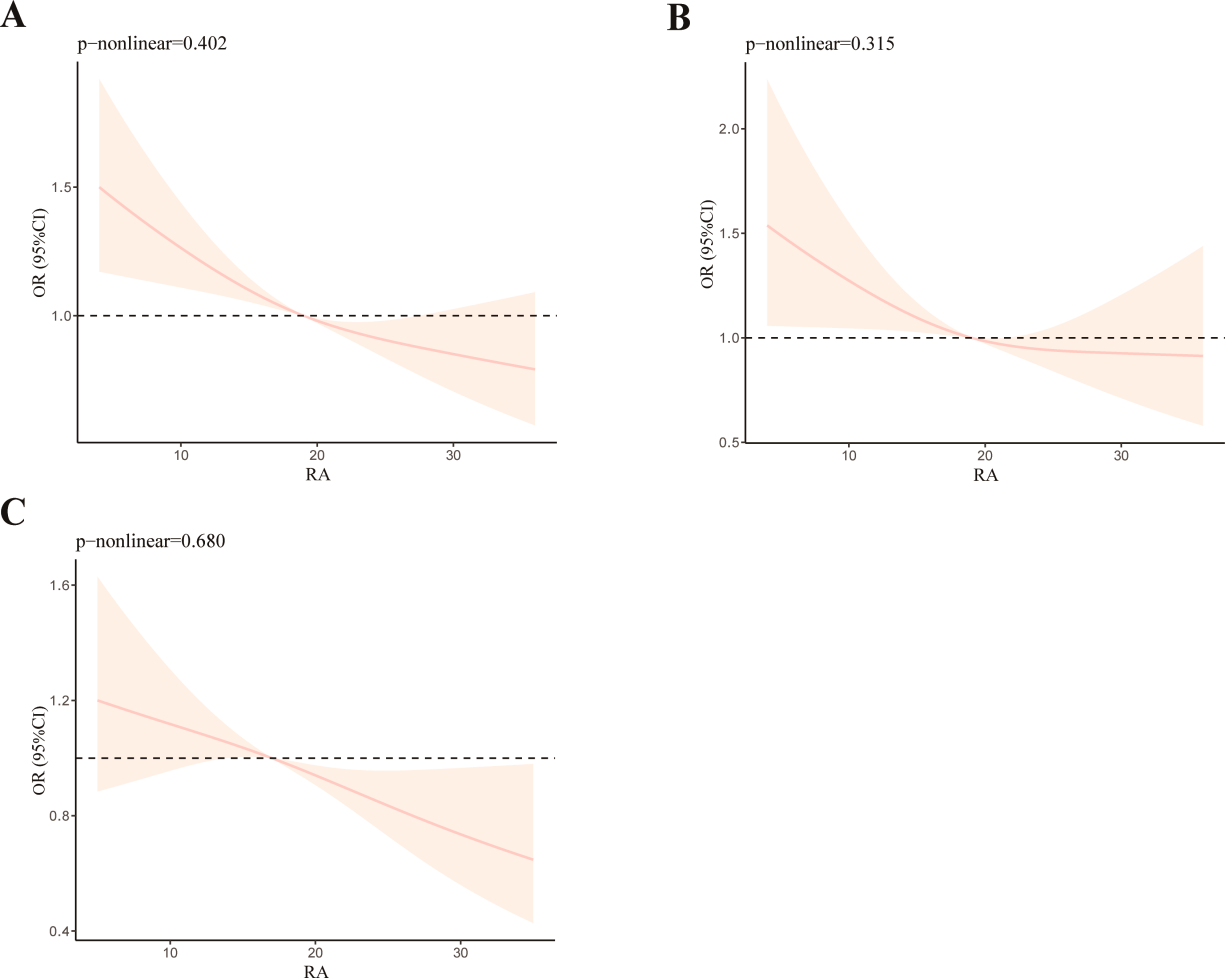
RCS analysis of OBS and RA **(A)**, RCS analysis of OBS and RA with participants aged from 20 to 60 **(B)**, and RCS analysis of OBS and RA with participants aged ≥60 **(C)**.

Restricted cubic spline (RCS) analysis was chosen to investigate the nonlinear association between OBS and RA. After modifying all covariates, we found that as the OBS value increases, the incidence of RA decreases (**Figure 3A**). This was consistent with our research findings mentioned above. Although the overall trend remains consistent, the nonlinear relationship between OBS and RA is not significant (*p*-nonlinear = 0.402). We also performed RCS analysis on different age groups: 20–60 (**Figure 3B**) and ≥60 (**Figure 3C**). We observed that the younger group (*p*-nonlinear = 0.315) exhibits a more pronounced nonlinear relationship compared to the older group (*p*-nonlinear = 0.680).

### 3.5 Sensitivity analysis

With the model adjusted for all covariates, sensitive analysis showed that no evident changes of outcomes between OBS and RA were observed after removing any component from the OBS. This indicated that our experimental results are robust. The concrete data could be found in **Supplementary Table S4**.

## 4 Discussion

Our study has innovatively discovered that a higher OBS has a protective effect on decreasing the odds of RA among individuals aged 20 or above. Individuals in Q2 demonstrated a higher degree of association compared to those in Q1, and the results remained unchanged after all covariates have been adjusted. Compared to their counterparts, OBS exhibited a more pronounced association with RA in younger, highly educated, married, and non-Hispanic White participants. However, a high level of OBS was not conducive to reduce the incidence of RA among unmarried participants. Individuals who suffered CKD or CVD showed a closer linkage between OBS and RA, but those with diabetes or liver disease displayed a weaker connection. Through RCS, the nonlinear relationship between OBS and RA was not significant. However, after conducting the subgroup RCS based on age, it was indicated that age might be a potential factor influencing the nonlinear relationship.

The risk of RA is closely associated with oxidative stress (4, 20, 21). Diet has long been considered by scientists as a risk factor for triggering RA (5, 22), and numerous studies have confirmed that consuming foods rich in antioxidant capacity can intervene in RA through mechanisms like anti-inflammatory (23) or gutting microbiota regulation (22). However, there are still many studies that questioned this assertion (6, 24–26). According to some cohort studies, vitamins, trace elements, and some dietary supplements from daily diets could potentially prevent the occurrence of RA in the long term (27, 28). These main components were adopted into OBS so as to evaluate the comprehensive effects clearly.

There was less controversy among scientists regarding the effects of physical activity, and proper exercise greatly prevents RA or improves the quality of life of RA patients (29). However, it seemed that BMI and smoking potentially influence the risk of RA with complex interactions which needed to be taken seriously (30). The impact of alcohol consumption on RA remained fully unclear, and there might be potential relationships with the frequency of drinking and the types of alcohol consumed (31). The impact of these lifestyle factors on RA, whether positive or negative, is incorporated into the final score presented by OBS. Previous research suggested that identifying the effects of oxidative stress-related exposures on health outcomes from different people is pretty challenging because each influential factor is often weak and interrelated (32). Therefore, as a comprehensive measurement, OBS includes both pro-oxidant and antioxidant dietary and lifestyle factors and appears more reasonable to reflect the interaction of pro- and anti-oxidant factors on oxidative stress-related health outcomes (33).

A higher OBS objectively reflects higher levels of antioxidant capacity in the body (34). Our research results are consistent with the existing mainstream findings. However, we noticed that in the models where adjustments were not fully made (model 1, model 2, and model 3), the relationship between OBS and RA appeared stronger in Q2 compared to the higher levels of OBS in Q3 and Q4. This demonstrated that the confounding disease factors, including CKD, diabetes, CVD, and liver disease, had interfered in the association between OBS and RA to some extent. According to a study by Hickson et al., individuals diagnosed with RA were more likely to suffer CKD than those without RA, and the corresponding risk of mortality also increased (35). It was widely recognized that individuals with RA bear a greater burden and faced a twofold-higher risk of CVD (36), and persistent inflammation was considered as the potential mechanism (37). Most existing studies demonstrated that patients with RA were prone to get a greater odd of liver disease, especially non-alcoholic fatty liver disease (NAFLD) (38, 39). A meta-analysis pointed out that one in every three RA patients is affected by NAFLD, which is nearly equivalent to the overall prevalence rate in the general population (39). Comparatively speaking, there were more varying opinions regarding the association between RA and diabetes (40, 41). However, the consensus from most rigorously conducted studies still suggested a link between them (42).

Existing studies had shown a gender correlation between OBS and health outcomes like diabetes (12), depression (13), and even sleep quality (16). However, this was not well reflected in our subgroup analysis. Generally, RA is divided into early-onset (EORA) and late-onset (LORA) types, with the threshold typically set at 60 years of age (43). We found that the association between OBS and EORA was stronger compared to LORA, and a more significant nonlinear relationship was also found in EORA. These could be attributed to the higher prevalence of LORA (44). Elderly persons also tended to have higher inflammatory parameters and weaker adaptability to oxidative stress (45). The characteristics or behaviors of the younger population, like coffee consumption (46, 47) and sleep loss (48, 49), lead to a more complex relationship, too. Therefore, intervening in RA by improving diet and lifestyle seems to be more effective for younger individuals. However, there was no definitive conclusion in the scientific community regarding the prognosis of EORA and LORA (44). By introducing OBS, we offered a fresh perspective for future research to analyze the age effects on RA.

It was worth mentioning that the subgroup of marital status showed a significant impact in our analysis. In the unmarried group, the positive influence from OBS is not evident after adjusting some common confounding factors. A review by Manfredini et al. explained that married individuals exhibited significantly better health outcomes compared to those who were unmarried (50). Married individuals may exhibit healthier dietary habits (51) and higher levels of physical activity (52) than single ones. These factors were precisely components of OBS. Moreover, previous studies indicated that marriage can decrease the risk of RA and slow down the progression of the disease (53), and it was said that social support played an important role in it (54). However, we noticed that a study by Reese et al. stated that it was more significant to think about the degree of adjustment in a marriage rather than merely whether or not one is married (55). The positive psychological implications of a good marital status can be greatly helpful for RA patients (55, 56).

Currently, the treatment of RA mainly relies on anti-inflammatory drugs and immunomodulators (57, 58). There is indeed controversy in the scientific community regarding the efficacy of antioxidants in the treatment of RA (59, 60). Some studies suggest that antioxidants may help reduce inflammation and oxidative stress, potentially improving RA (61, 62). However, other research indicate that their effect on RA may be limited (60, 63). By utilizing OBS and integrating people’s daily diet and lifestyle, we hope to provide a new approach for the prevention, treatment, and prognosis of RA through a series of routine measures.

Our research holds some advantages, namely: (1) we have integrated data from the NHANES database over the past decade, providing a sufficient sample size and population representativeness; (2) we utilized an integrated OBS incorporating diet and lifestyle factors for RA and found a negative correlation between them, which provided a more comprehensive and objective direction for future researchers in this field; (3) we adjusted for numerous confounding factors and employed various analytical methods to demonstrate the reliability of our findings; and (4) we discovered that OBS has a more significant protective effect on younger, married individuals, while it exhibits an opposite effect on the unmarried population.

There still some disadvantages that exist in our study, namely: (1) limited by the cross-sectional study, we were unable to ascertain a causal correlation between OBS and RA; (2) OBS consists of diet component and lifestyle component—we did not separately analyze and discuss the potential mechanisms of each one; and (3) although there are differences among subgroups, these differences are not highly significant. Thus, further research are needed to corroborate our findings.

## 5 Conclusion

In conclusion, a protective effect was discovered between OBS and RA among participants from NHANES. This correlation was more obvious in younger as well as married individuals. In conducting a subgroup analysis of common diseases including CKD, diabetes, CVD, and liver disease, OBS presents a different extent to its impact on RA. By taking into account individual differences such as age and marital status, we hope to provide personalized approaches for the prevention of RA. As a method closer to the general public and one that is easier to calculate, OBS will be expected to offer its unique value for the diagnosis and treatment of RA in the future.

## Data Availability

Publicly available datasets were analyzed in this study. This data can be found here: NHANES.
