# Supplementary material for: Oxidative balance score and the potential for suffering rheumatoid arthritis: a cross-sectional study
Source: Front Immunol. 2024 Nov 1;15:1454594. doi: 10.3389/fimmu.2024.1454594 (PMC11563821; doi:10.3389/fimmu.2024.1454594)
Supplement: Supplementary file 1 [file DataSheet1.docx]

**Table S1.** Composition oxidative balance score (OBS)

| Dietary components | |  | Lifestyle components | |
| --- | --- | --- | --- | --- |
| Name | Property |  | Name | Property |
| Dietary fiber | Antioxidant |  | Physical activity | Antioxidant |
| Carotene | Antioxidant |  | Alcohol | Pro-oxidant |
| Riboflavin | Antioxidant |  | Body mass index | Pro-oxidant |
| Niacin | Antioxidant |  | Cotinine | Pro-oxidant |
| Vitamin B6 | Antioxidant |  |  |  |
| Total folate | Antioxidant |  |  |  |
| Vitamin B12 | Antioxidant |  |  |  |
| Vitamin C | Antioxidant |  |  |  |
| Vitamin E | Antioxidant |  |  |  |
| Calcium | Antioxidant |  |  |  |
| Magnesium | Antioxidant |  |  |  |
| Zinc | Antioxidant |  |  |  |
| Copper | Antioxidant |  |  |  |
| Selenium | Antioxidant |  |  |  |
| Total fat | Pro-oxidant |  |  |  |
| Iron | Pro-oxidant |  |  |  |

**Table S2.** Description of covariates assessment.

| Covariates | Detailed covariate information |
| --- | --- |
| Age | 20-60 and ≥60 |
| Sex | Male and female |
| Race | Five groups: Mexican American, other Hispanic, non-Hispanic white, non-Hispanic black,  and others |
| Education | Three groups: less than 9th grade/9-11th grade (Includes 12th grade with no diploma), high school graduate/GED or equivalent and some college or AA degree/College graduate or above |
| Marital | Three groups: married/Living with Partner, widowed/Divorced/Separated and never married |
| PIR | Three groups: ≤1.3 (low), 1.3-3.5 (mediate) and ＞3.5 (high) |
| Kidney disease | Urine albumin/creatinine ratio >30 mg/g or estimated eGFR ≤60ml/min/1.73 m2 was diagnosed as chronic kidney disease. |
| Diabetes | Self-reported diagnosis by a doctor and ongoing oral treatment with diabetes medication. |
| Cardiovascular disease | Self-reported diagnosis included five independent diseases: heart failure, coronary heart disease, angina, heart attack and stroke. |
| Liver disease | Self-reported diagnosis by being informed liver condition. |

**Table S3.** Characteristics of participants aged 20-80 by OBS quantiles in the U.S.

| **Characteristics** | **Overall**  **(n = 21,415)** | **Q1, [3,12]**  **(n = 4,938)** | **Q2, (12,18]**  **(n = 5,544)** | **Q3, (18,24]**  **(n = 5,348)** | **Q4, (24,37]**  **(n = 5,585)** | ***p* value** |
| --- | --- | --- | --- | --- | --- | --- |
| **Age, n (%)** | 48.7±17.4 | 50.0±17.6 | 49.9±17.7 | 48.4±17.3 | 46.6±16.8 | <0.001 |
| 20-60 | 14,705 (68.7) | 3,179 (64.4) | 3,643 (65.7) | 3,709 (69.4) | 4,174 (74,7) |  |
| ≥60 | 6,710 (31.3) | 1,759 (35.6) | 1,901 (34.3) | 1,639 (30.6) | 1,411 (25.3) |  |
| **Sex, n (%)** |  |  |  |  |  | <0.001 |
| Male | 10,432 (48.7) | 2,408 (48.8) | 2,724 (49.1) | 2,597 (48.6) | 2,703 (48.4) |  |
| Female | 10,983 (51.3) | 2,530 (51.2) | 2,820 (50.9) | 2,751 (51.4) | 2,882 (51.6) |  |
| **Race,** **n (%)** |  |  |  |  |  | <0.001 |
| Mexican American | 2,760 (12.9) | 457 (9.3) | 690 (12.4) | 726 (13.6) | 887 (15.9) |  |
| Other Hispanic | 2,125 (9.9) | 447 (9.1) | 568 (10.2) | 533 (10.0) | 577 (10.3) |  |
| Non-Hispanic White | 8,413 (39.3) | 1,830 (37.1) | 2,171 (39.2) | 2,144 (40.1) | 2,268 (40.6) |  |
| Non-Hispanic Black | 4,729 (22.1) | 1,614 (32.7) | 1,251 (22.6) | 1,020 (19.1) | 844 (15.1) |  |
| Other Race | 3,388 (15.8) | 590 (11.8) | 864 (15.6) | 925 (17.2) | 1,009 (18.1) |  |
| **Education, n (%)** |  |  |  |  |  | <0.001 |
| Below high school | 3,937 (18.4) | 1,130 (22.9) | 1,065 (19.2) | 904 (16.9) | 838 (15.0) |  |
| High school | 4,840 (22.6) | 1,350 (27.3) | 1,338 (24.1) | 1,128 (21.1) | 1,024 (18.3) |  |
| Above high school | 12,638 (59.0) | 2,458 (49.8) | 3,141 (56.7) | 3,316 (62.0) | 3,723 (66.7) |  |
| **Marital status, n (%)** |  |  |  |  |  | <0.001 |
| Married/cohabiting | 12,833 (59.9) | 2,613 (52.9) | 3,319 (59.9) | 3,341 (62.5) | 3,560 (63.7) |  |
| Widowed/divorced/separated | 4,446 (20.8) | 1,236 (25.0) | 1,227 (22.1) | 1,051 (19.7) | 932 (16.7) |  |
| Never married | 4,136 (19.3) | 1,089 (22.1) | 998 (18.0) | 956 (17.8) | 1,093 (19.6) |  |
| **PIR, n (%)** | 2.0±0.8 | 1.8±0.8 | 2.0±0.8 | 2.0±0.8 | 2.1±0.8 | <0.001 |
| ≤1.3 | 6,531 (30.5) | 1,925 (39.0) | 1,702 (30.7) | 1,453 (27.2) | 1,451 (26.0) |  |
| 1.3-3.5 | 8,083 (37.7) | 1,883 (38.1) | 2,150 (38.8) | 2,062 (38.6) | 1,988 (35.6) |  |
| >3.5 | 6,801 (31.8) | 1,130 (22.9) | 1,692 (30.5) | 1,833 (34.2) | 2,146 (38.4) |  |
| **CKD, n (%)** |  |  |  |  |  | <0.001 |
| Yes | 5,775 (27.0) | 1,695 (34.3) | 1,588 (28.6) | 1,313 (24.6) | 1,179 (21.1) |  |
| No | 15,640 (73.0) | 3,243 (65.7) | 3,956 (71.4) | 4,035 (75.4) | 4,406 (78.9) |  |
| **Diabetes, n (%)** |  |  |  |  |  | <0.001 |
| Yes | 2,147 (10.0) | 556 (11.3) | 634 (11.4) | 505 (9.4) | 452 (8.1) |  |
| No | 19,268 (90.0) | 4,382 (88.7) | 4,910 (88.6) | 4,843 (90.6) | 5,133 (91.9) |  |
| **CVD, n (%)** |  |  |  |  |  | <0.001 |
| Yes | 2,207 (10.3) | 749 (15.2) | 621 (11.2) | 481 (9.0) | 356 (6.4) |  |
| No | 19,208 (89.7) | 4,189 (84.8) | 4,923 (88.8) | 4,867 (91.0) | 5,229 (93.6) |  |
| **Liver disease, n (%)** |  |  |  |  |  | <0.001 |
| Yes | 928 (4.3) | 211 (4.3) | 205 (3.7) | 248 (4.6) | 264 (4.7) |  |
| No | 20,487 (95.7) | 4,727 (95.7) | 5,339 (96.3) | 5,100 (95.4) | 5,321 (95.3) |  |
| Note: Continuous variables were presented as mean ± standard deviation (SD); categorical variables were presented as n (%). The OBS was divided into four levels by quartile (3 ≤ Q1 ≤ 12, 12 < Q2 ≤ 18, 18 < Q3 ≤ 24, and 24 < Q4 ≤37). OBS: oxidative balance score; PIR: family poverty-income ratio CKD: chronic kidney disease; CVD: cardiovascular disease | | | | | | |

**Table S4.** Sensitivity analysis for the association of OBS with RA.

| **Condition** | **OR (95% CI)** | ***p* value** |
| --- | --- | --- |
| Dietary fiber excluded | 0.98 (0.97, 0.99) | <0.001 |
| Carotene excluded | 0.98 (0.97, 0.99) | <0.001 |
| Riboflavin excluded | 0.98 (0.97, 0.99) | <0.001 |
| Niacin excluded | 0.98 (0.97, 0.99) | <0.001 |
| Vitamin B6 excluded | 0.98 (0.97, 0.99) | <0.001 |
| Total folate excluded | 0.98 (0.97, 0.99) | <0.001 |
| Vitamin B12 excluded | 0.98 (0.97, 0.99) | <0.001 |
| Vitamin C excluded | 0.98 (0.97, 0.99) | <0.001 |
| Vitamin E excluded | 0.98 (0.97, 0.98) | <0.001 |
| Calcium excluded | 0.98 (0.97, 0.99) | <0.001 |
| Magnesium excluded | 0.98 (0.97, 0.99) | <0.001 |
| Zinc excluded | 0.98 (0.97, 0.99) | <0.001 |
| Copper excluded | 0.98 (0.97, 0.99) | <0.001 |
| Selenium excluded | 0.98 (0.97, 0.99) | <0.001 |
| Total fat excluded | 0.98 (0.97, 0.99) | <0.001 |
| Iron excluded | 0.98 (0.97, 0.99) | <0.001 |
| Alcohol excluded | 0.98 (0.97, 0.99) | <0.001 |
| Body mass index excluded | 0.98 (0.97, 0.99) | <0.001 |
| Cotinine excluded | 0.98 (0.97, 0.99) | <0.001 |
| Physical activity excluded | 0.98 (0.97, 0.99) | <0.001 |


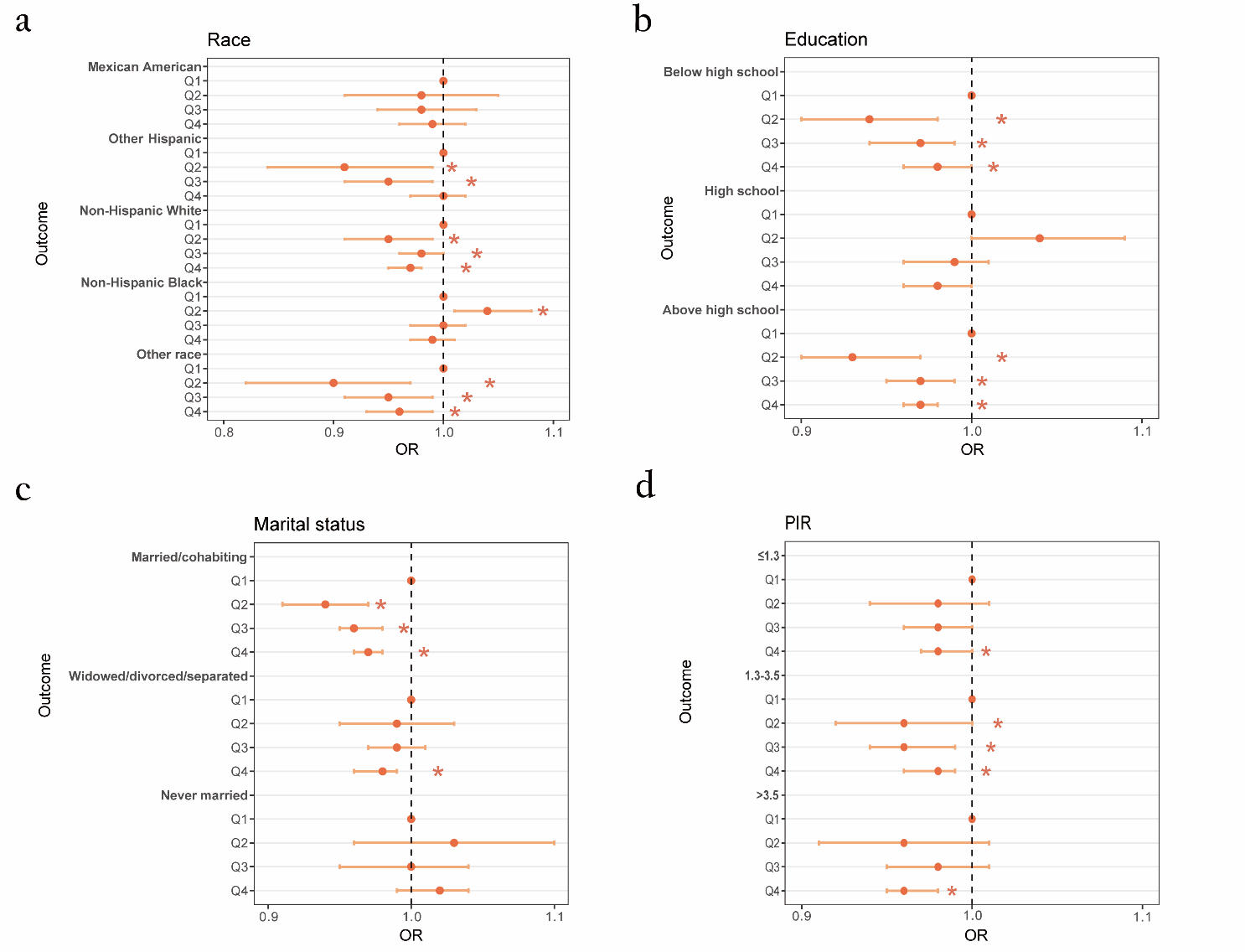


**Figure S1.** Forest plot of the race subgroup (a); Forest plot of the education subgroup (b); Forest plot of the marital status subgroup (c); Forest plot of the PIR subgroup (d)
